# G-Quadruplex-Binding Proteins: Promising Targets for Drug Design

**DOI:** 10.3390/biom12050648

**Published:** 2022-04-29

**Authors:** Huiling Shu, Rongxin Zhang, Ke Xiao, Jing Yang, Xiao Sun

**Affiliations:** State Key Laboratory of Bioelectronics, School of Biological Science and Medical Engineering, Southeast University, Nanjing 210096, China; 230228565@seu.edu.cn (H.S.); rongxinzhang@outlook.com (R.Z.); kexiao@seu.edu.cn (K.X.); 220195127@seu.edu.cn (J.Y.)

**Keywords:** G-quadruplex, G-quadruplex-binding protein, drug target, G4, diseases, therapeutics

## Abstract

G-quadruplexes (G4s) are non-canonical secondary nucleic acid structures. Sequences with the potential to form G4s are abundant in regulatory regions of the genome including telomeres, promoters and 5′ non-coding regions, indicating they fulfill important genome regulatory functions. Generally, G4s perform various biological functions by interacting with proteins. In recent years, an increasing number of G-quadruplex-binding proteins have been identified with biochemical experiments. G4-binding proteins are involved in vital cellular processes such as telomere maintenance, DNA replication, gene transcription, mRNA processing. Therefore, G4-binding proteins are also associated with various human diseases. An intensive study of G4-protein interactions provides an attractive approach for potential therapeutics and these proteins can be considered as drug targets for novel medical treatment. In this review, we present biological functions and structural properties of G4-binding proteins, and discuss how to exploit G4-protein interactions to develop new therapeutic targets.

## 1. Introduction

G-quadruplex (G4) is a polymorphic and stable secondary nucleic acid structure which is mostly formed in single-stranded DNA or RNA guanine-rich regions [1,2,3]. The basic structural unit of the G4 is the G-quartet, a square planar assembly of four guanines held together by Hoogsteen hydrogen bonding [4]. Stable G4 structure formation is driven by stacks of 2–4 G-quartets in general and monovalent cations such as Na^+^ and K^+^ in the central channel of G4 helix. G4s could be assembled from the same (intramolecular) or distinct (intermolecular) G-rich strands. G4 structures may fold into diverse topologies, which are dictated by the numbers of stacked G-quartets, the orientation and polarity of the nucleic acid strands and the glycosidic conformation of guanine bases in quartets [3,4,5]. For example, DNA G4 can form a parallel, anti-parallel or mixed structure, while RNA G4 is more inclined to form a parallel structure and it has higher thermal stability [6,7].

**Figure 1:**
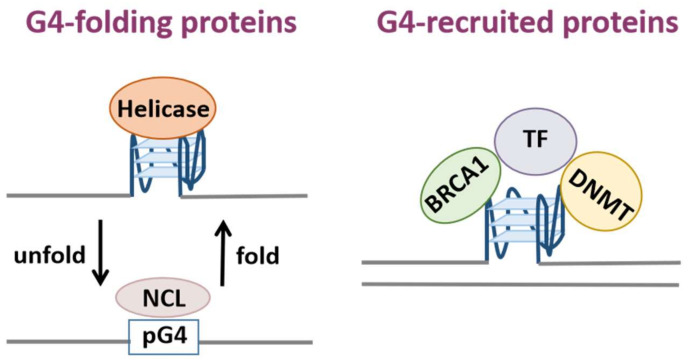
Classification of G-quadruplex-binding proteins. G4BPs can be classified into two categories: G4-folding proteins which can fold or unfold G4s such as nucleolin (NCL) and helicases and G4-recruited proteins which can be recruited by G4 such as transcription factors (TF), DNA repair proteins (BRAC1) and chromatin remodeling proteins (DNA methyltransferase, DNMT).

**Figure 2:**
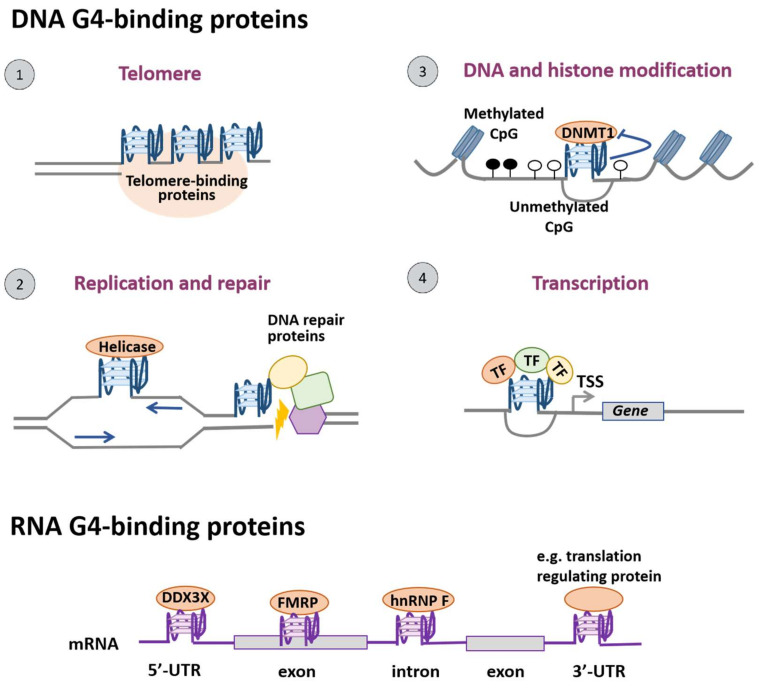
Location and biological functions of G-quadruplex-binding proteins. According to their location, G4BPs can be classified into the following two categories: DNA and RNA G4BPs. In cells, G4BPs perform various biological functions. ① At telomeres, telomere-binding proteins form a ternary complex with the G4 structures of telomeric DNA for telomere maintenance. ② G4s formed during replication present obstacles to the replication machinery and should be resolved by helicases for effective replication. Meanwhile, DNA repair proteins could be recruited to G4 sites to repair DNA double-strand breaks (DSB). ③ In terms of epigenetic control, the inactivation of DNA methyltransferase 1 (DNMT1) when binding G4s results in hypomethylation at CpG islands. ④ In the promoter regions, multiple transcription factors can bind G4s upstream of the transcription start site (TSS) and activate gene transcription. RNA G4BPs regulate mRNA maturation including mRNA export and splicing, and they also function in translational control.

Researchers are able to obtain sequence information with the potential to form G4 structures with an algorithmic analysis of G4 motifs such as G_X_N_1-7_G_X_N_1-7_G_X_N_1-7_G_X_N_1-7_ (x ≥ 3 and N can be any base) [8,9]. However, further experimental approaches are required in order to verify whether these sequences truly form G4 structures. Biophysical methods are commonly used, such as circular dichroism (CD), ultraviolet melting, nuclear magnetic resonance (NMR) spectroscopy and X-rays [3,10,11,12]. Furthermore, a number of genomic techniques have been developed in recent years, including genome-wide DNA polymerase-stop assay followed by high-throughput sequencing (G4-seq), chromatin immunoprecipitation followed by high-throughput sequencing using BG4 (G4 ChIP-seq) or the use of a small artificial protein G4 probe (G4P) [1,3,13,14,15]. Notably, the latest technique Cleavage Under Targets and Tagmentation (CUT&Tag) for mapping native G4 in mammalian cells can be applied to single-cell G4 detection [16]. In addition, RNA G4 in the human transcriptome can be detected using reverse transcriptase stalling with next-generation sequencing (rG4-seq) and G4-RNA-specific precipitation (G4RP) with sequencing (G4RP-seq) [17,18]. The distribution information of G4s in the genome and transcriptome also lays a solid foundation for subsequent research on the functions of G4s. Sequences with the potential to form G4 structures (pG4) are present in the genomes of all organisms [5,19]. The location of pG4s is not random in that pG4s occur primarily in the functional regions of the genome and they are highly conserved among different species [5]. Significantly, it was found that pG4s are highly enriched in regulatory regions such as telomeres, gene promoters and the border between introns and exons. Furthermore, recent studies have revealed that 90% of human DNA replication origins contain pG4 motifs, and they also colocalize with the region-specifying 5′-untranslated region (UTR) of the encoded mRNAs among 3000 human genes [5,20]. It can be seen that G4 structures correlate closely with the functions of the genome, and play an important role in DNA replication, transcription, translation, and epigenetic modification. The participation of proteins is necessary for the formation of G4 structures and the fulfillment of their biological functions. These proteins that can specifically bind to G4s are generally referred to as G-quadruplex-binding proteins (G4BPs). Firstly, G4BPs could be classified into two main types according to their control mechanisms and functional relationships with G4s, i.e., G4-folding proteins which have an effect on G4 structures and G4-recruited proteins which are functional proteins recruited by G4 (Figure 1). Secondly, G4BPs can also be divided into the following two categories based on the distribution of G4s in the genome: DNA and RNA G4BPs (Figure 2). To be specific, the structural effects of G4-folding proteins on the G4s lie in two aspects. On one hand, these proteins may promote pG4 to form a stable quadruplex structure; on the other hand, they have the ability to unfold G4s [21,22,23]. Furthermore, the fulfillment of the biological functions of G4 structures is generally accompanied by the recruitment of G4BPs [10]. Telomere-binding proteins can bind to G4s and unfold these structures at telomeres to maintain the length and integrity of telomeres [24,25]; various helicases and DNA repair proteins recruited to the G4 formation sites unwind G4 structures and remove DNA lesions to ensure efficient DNA replication and maintain genome integrity [21,22,26]; epigenetic modulators regulate the methylation of DNA and histones through their interaction with G4s [27,28]; transcription factors bind to G4s at the promoter sites to aid or suppress gene transcription [29,30]. In addition, G4BPs are also involved in multiple biological processes such as post-transcriptional processing, mRNA maturation and translational regulation (Table 1).

Therefore, the formation, stabilization and resolution of G4 structures in vivo should be strictly regulated. A critical step to activate or inactivate physiological or pathological pathways is the recognition and processing of G4s by G4BPs [50]. Analyzing the amino acid composition and the structural properties of G4BPs offers explanation of G4 recognition mechanisms and also provides a structural basis for drug design based on G4BPs [51,52]. The extensive research on spatiotemporal G4BP expression will provide new targets for drug design and pave the way for novel cancer therapy [53,54]. However, there are some difficulties in the analysis of G4-protein recognition. Firstly, the interaction between G4s and proteins in vitro may not confirm the existence of such an event in vivo due to the plasticity of G4 formation and distinct cellular contexts. Secondly, insufficient three-dimensional structures of G4-protein complexes could be an obstacle to molecular modeling and rational targeting [55]. In this review, we first briefly introduce tools for the detection of G4BPs, then systematically describe DNA and RNA G4BPs and their regulatory roles. We also analyze the structural properties of these proteins in detail and summarize their relationships with multiple diseases. Finally, we discuss how to exploit G4-protein interactions for drug target identification and drug design, thus opening the way to the development of novel therapeutics.

## 2. G-Quadruplex-Binding Proteins

### 2.1. Detection of G-Quadruplex-Binding Proteins

The G4 structure is highly dynamic in vivo and depends on the cell type and chromatin state [2]. Meanwhile, the formation and unwinding of G4 structures across the whole genome and transcriptome are directly or indirectly regulated by G4BPs, thereby affecting various biological processes [2,3]. Thus, the identification and an in-depth study of G4BPs can provide a full explanation of G4–protein interactions and their biological roles in vivo. These studies will further inspire the development of medical applications for these proteins.

G4BPs are most often identified by biochemical experiments. The commonly used methods include affinity chromatography, quantitative methods based on mass spectrometry, and fluorescence energy resonance transfer (FRET) technology. Affinity chromatography is often used in combination with mass spectrometry to separate proteins that bind to specific G4 motifs [56]. For instance, this method was applied to the identification of proteins binding to G4s in the 5’ UTR of tumor-associated mRNA [57]. FRET is a spectroscopic technique that provides information about the conformation and dynamics of biomolecules. It has been widely used because this technology can detect whether there is a direct interaction between G4 structures and proteins in vivo [58,59]. In addition to performing biochemical experiments, computational analyses could be conducted to identify G4BPs. For example, putative G4 motifs can be predicted at known binding sites of nucleic acid-binding proteins, or the computational modeling of structural features may be exploited to discover new G4BPs [2,51,52,60].

Recently, great advances have been made in the identification of G4BPs. The affinity purification experiments do not take into consideration the native chromatin state, so Shankar Balasubramanian et al. pioneered a co-binding-mediated protein profiling (CMPP) approach for the exploration of DNA G4BPs in living cells [61]. Researchers designed small-molecule ligands that specifically target DNA G4 in cells so that the probes could approach G4BPs with minimal interference with G4-protein interactions and enable labeling by subsequent photoproximity crosslinking [61]. The strategy was employed to identify hundreds of potential G4BPs, and finally in vitro experiments confirmed the binding specificity of several candidate proteins. Overall, this approach laid the foundations for the subsequent investigation of new G4BPs.

In conclusion, the detection methods are made up of in vivo, in vitro and in silico approaches. Generally, in vivo and in silico approaches are employed to identify potential G4BPs while in vitro approaches are utilized to confirm G4-protein interactions.

### 2.2. DNA G-Quadruplex-Binding Proteins

Recent discoveries related to the involvement of DNA G4BPs in the regulation of cellular fundamental functions will be discussed in the following section. 

#### 2.2.1. Telomeric G-Quadruplex-Binding Proteins

The human telomeric sequence was one of the first sequences discovered to form G4 structures. Telomeres are nucleoprotein complexes that constitute the ends of eukaryotic chromosomes, which play crucial roles in maintaining the integrity and stability of the genome [10,62]. When specific proteins bind to telomeric DNA, these proteins can prevent not only the degradation of the chromosome ends by nucleases, but also the recognition of them as broken fragments by the DNA repair mechanism [24,25,62].

Telomeric DNA is highly conserved between vertebrates and consists of the identical TTAGGG short repeat sequences with a guanine-rich single-stranded 3′ overhang [62]. These repeat sequences have the potential to form a G4 structure. The experiments using G4 specific antibodies and G4 ligands also confirmed the existence of G4 structures at telomeres in vivo [10].

**Figure 3:**
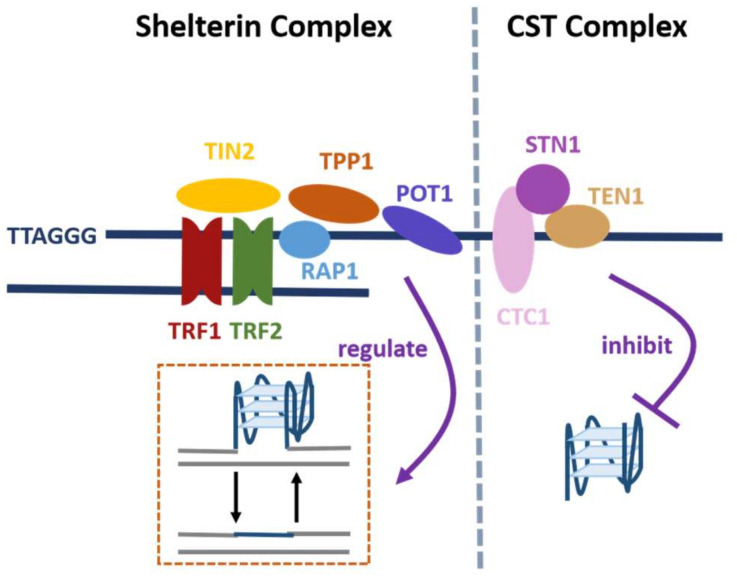
Schematic diagram of the telomere-associated protein complexes shelterin and CST. Shelterin and CST play crucial roles in telomere maintenance. TPP1-POT1 subunit of shelterin regulates the folding and unwinding of G4 structures. CST could resolve and prevent the formation of G4 structures.

Mammalian telomeric DNA is bound by a protein complex called shelterin, which protects the DNA termini from being considered as damaged and prevents the triggering of the repair mechanism (Figure 3) [31,63,64]. The proteins TRF1 and TRF2 (Telomere Repeat Binding Factor 1 and 2) in shelterin bind to double-stranded telomeric DNA; POT1 (Protection of Telomeres protein 1) binds to 3′ overhang of telomeric repeats and regulates the folding and unwinding of the G4 structures with its heterodimeric partner TPP1 (TIN2 Interacting Protein) [10,65,66,67]. TPP1 connects POT1 to TRF1 and TRF2 via TIN2 (TRF1-interacting Nuclear protein 2) [34]. In addition, the study found that the helicases WRN (Werner syndrome ATP-dependent helicase) and BLM (Bloom syndrome protein) of the RecQ family are recruited to the telomeres and unfold the G4 structures to maintain the integrity of telomeres and ensure telomere replication [68,69]. WRN colocalizes with TRF2 and POT1, and both WRN and BLM can bind to POT1 with high affinity, which indicates that the telomeric DNA-binding proteins are essential for the recruitment of helicases [5].

Mammalian cells also contain another telomere-associated protein complex called CST (CTC1-STN1-TEN1), which plays a crucial role in efficient telomere replication and in the maintenance of telomere length (Figure 3) [34,70,71,72]. Human CST is a single-stranded DNA-binding protein complex that helps to solve the genome-wide replication problems [34,71]. For example, GC-rich regions of the genome may induce obstacles to DNA replication, because DNA polymerase may stall at a G4. Experiments confirmed that CST could bind to the G4s and unfold them [34]. G4 structures possibly form on the lagging strand template at telomeres where WRN, BLM and POT1 all participate in G4 removal [5,34,73,74]. However, the presence of CST could make the replication of double-stranded telomeric DNA more effective as this complex unwinds G4 structures more rapidly than POT1 [34].

Other telomere-binding proteins also have similar functions as is the case for these two protein complexes. For example, heterogenous nuclear ribonucleoprotein (hnRNP) A1 and hnRNP A2/B1 form macromolecular complexes with telomere-maintaining factors to regulate telomere length [75]. Given that hnRNP A1 has the ability to unwind the telomeric G4 structures, it can stimulate telomere elongation through the resolution of G4 structures at the end of telomeres [76,77].

#### 2.2.2. G-Quadruplex-Binding Proteins Involved in Replication

The G4 structure has a dual effect on the process of DNA replication. On one hand, the G4 structure has been demonstrated to support the initiation of DNA replication at the replication origin [78]. Furthermore, the G4 structure may prevent the uncoupling of the leading- and lagging-strand polymerases, thereby protecting proper replication [22]. On the other hand, the G4 could hinder the progression of the replication fork and influence DNA synthesis, which may lead to mutations and deletions in the genome. Consequently, helicases usually unfold the G4 structures before replication to maintain genome stability [22].

FANCJ (Fanconi anemia complementation group J) is a 5′–3′ DNA helicase, which is involved in various biological processes such as DNA damage repair, G4 resolution, homologous recombination and genome stability maintenance [23]. FANCJ can unfold and remove G4 structures for efficient DNA replication while its absence will stop replication at G4s and eventually lead to DNA damage [79]. Studies have shown that FANCJ might promote replication at G4s by two independent mechanisms [80]. One mechanism is that FANCJ may cooperate with polymerase REV1 to aid replication at the replication fork [81]. REV1 destabilizes the G4 structures so that FANCJ can unwind them from the other side of the G4 structures. Second, WRN or BLM may assist FANCJ to bind and unfold the G4s from the opposite direction in order to promote replication synergistically [21,80,82].

The helicase Pif1 from yeasts is able to bind and unfold G4 structures to support DNA replication. It is not clear whether Pif1 can play a role unwinding G4 structures on both chains or if it has a binding preference for the G4 structure at a certain chain [22]. However, recent studies have suggested that the ubiquitin ligase complex protein Mms1 is not only a DNA G4-binding protein, but also assists Pif1 to bind to a specific G4 structure located on the lagging strand. It could be observed that the absence of Mms1 leads to a reduction in Pif1 binding and slow replication at G4 motifs, and finally causes G4-dependent genome instability [83].

#### 2.2.3. G-Quadruplex-Binding Proteins Involved in Transcription

It has been found that about 50% of human genes contain G4 motifs near their promoter region, which indicates that G4s play an essential role in the regulation of gene expression [5]. When DNA G4 is located at the first intron downstream of the transcription start site (TSS), it blocks the RNA polymerase and suppresses transcription [29]. However, recent studies have shown that endogenous G4s in promoters are prominent binding sites for multiple transcription factors and are thus invariably linked to high transcription levels [29,30]. Notably, G4s and their associated transcription factors cooperate to shape the cell-specific transcriptome [29,84]. In fact, transcription factors account for a significant part of the G4BPs. Statistically, there are 14 transcription factors among the 56 DNA G4-binding proteins in the G4IPDB (G4 Interacting Proteins DataBase) [85]. For example, SP1 (Specificity protein 1) is a zinc finger transcription factor, which can bind to the G4 structures on the *c-KIT* promoter and regulate the expression of a variety of housekeeping genes [29]. MAZ (Myc-associated zinc finger) and PARP-1 (Poly [ADP-ribose] polymerase 1) interact with the G4 structures upstream of the transcription start site of *KRAS*, and both of them are activators of *KRAS* [2,5,86].

The G4 motif occurs more frequently in proto-oncogenes and regulatory genes than in housekeeping genes and tumor suppressor genes [5,87,88]. The first reported G4 on the promoter is formed in the nuclease hypersensitivity element III1 (NHE III1) which locates upstream of the P1 promoter of the proto-oncogene *c-MYC* [23,89]. This guanine-rich region controls 85–90% of the transcriptional activation of the gene, and can fold into an intramolecular parallel G4 as a transcriptional repressor element [76]. In addition to *c-MYC*, many genes have been demonstrated to form G4 structures in the promoter regions, such as proto-oncogenes *VEGF* [90], *KRAS* [91], *BCL-2* [92] and *c-KIT* [93]; human platelet-derived growth factor receptor *PDGFR-β* [94]; human telomerase reverse transcriptase *hTERT* [95] and other genes [23]. In particular, the G4s in the promoter regions of the proto-oncogenes have been most intensively studied so far [2].

Nucleolin (NCL) is a multifunctional phosphoprotein that is most abundant in the nucleolus. Nucleolin is mainly associated with ribosome biosynthesis and also involved in chromatin remodeling, transcriptional regulation, G4 binding and apoptosis [76]. Nucleolin can bind to the *c-MYC* G4 with high affinity and promote the formation and stabilization of G4 structures. The luciferase assay results also proved that the overexpression of nucleolin could contribute remarkably to a reduction in *c-MYC*-driven transcription [76]. Another protein NM23-H2 which belongs to the NM23 family of nucleoside diphosphate kinase (NDPK) has a completely different structure effect on G4s from nucleolin. It has a variety of functions, including kinase activity, promoter binding, transcriptional regulation and DNA repair [43]. Experiments have confirmed that NM23-H2 could bind to the *c-MYC* G4 to promote the unfolding of the G4 structure, thereby activating the transcription of *c-MYC* [43].

The tumor suppressor protein p53 functions in apoptosis, DNA repair, cell cycle regulation and aging. As a transcriptional regulator, p53 can inhibit the expression of cell cycle regulatory and growth promoting genes via multiple mechanisms and plays a key role in tumor suppression [44]. Previous studies have found that wild-type p53 (wtp53) and several types of mutant p53 (mutp53) have the ability to selectively bind *c-MYC* and *hTERT* promoter G4s [96], and the C-terminal region of p53 is essential for the recognition of the G4. Accordingly, the interaction between p53 and G4 structures in promoter regions of p53 target genes may play an important role in p53-mediated transcriptional regulation [44].

#### 2.2.4. Other DNA G-Quadruplex-Binding Proteins

Direct evidence has demonstrated that the endogenous human G4 DNA landscape is dynamically shaped by chromatin relaxation or cell status [14,97]. Indeed, several G4BPs also function in chromatin structure regulation and histone modification [98,99]. For example, various epigenetic and chromatin remodeling enzymes bind selectively to DNA G4 [2]. Genomic binding sites of the chromatin remodeling protein ATR-X colocalize with GC-rich tandem repeats and CpG islands (CGI) that have the potential to form G4 structures [100,101].

Guanine-rich sequences are very common around CpG islands, with a high distribution rate of up to 80% [23,102,103]. The presence of G4 structures is closely related to the hypomethylation of CpG islands in the human genome. Studies have revealed that DNMT1 (DNA methyltransferase 1) interacts with these G4 sites, which is consistent with the results observed in biophysical experiments. Specifically, DNMT1 shows a higher binding affinity to G4 compared with double-stranded, single-stranded or hemimethylated DNA [28]. Biochemical analyses demonstrated that G4 structures inhibit the enzymatic activity of DNMT1, and the formation of G4 also hinders DNMT1 to protect specific CpG islands from methylation and inhibit local methylation [28].

In addition, it has been found that G4s colocalize with CTCF (CCCTC-binding factor) binding sites in CpG islands and interact with CTCF in vitro. G4 is also crucial to the localization of CTCF [104]. CTCF is frequently recruited to CpG islands that are usually hypomethylated. Furthermore, the enrichment of G4s at CpG islands maintains CGI hypomethylation, which may explain the correlation between CpG islands and CTCF [104]. CTCF also functions as a chromatin remodeling factor with the capability of nucleosome repositioning; therefore, G4 can facilitate the binding of CTCF to genomic DNA by recruiting chromatin proteins [99].

### 2.3. RNA G-Quadruplex-Binding Proteins

It is easier for single-stranded RNA to form G4s in guanine-rich regions, and G4 is also an important structural characteristic of mRNA [10,46]. Recently, in vitro experiments combining high-throughput sequencing with reverse transcriptase stalling at RNA G4s (rG4) have found more than 13,000 loci with the potential to form rG4 structures in the human transcriptome; and immunofluorescence using G4 specific antibodies demonstrated rG4 formation in cells [17,105]. Notably, the highest abundance of rG4 is in functional regions including 5’ and 3’-UTR [46]. All these observations of the enrichment of rG4s in functionally important regions suggest that they play crucial roles in transcription termination, alternative splicing, translational regulation, and chromosome integrity maintenance [57,106].

A substantial number of proteins interacting with rG4s have been identified by biochemical experiments, for example hnRNPs, ribosomal proteins and splicing factors [10,57]. Although there are DNA and RNA G4 specific proteins, their binding proteins have a significant overlap due to structural similarities between DNA and RNA G4s [10]. Basically, the discrimination between DNA and RNA G4BPs may depend on their different biological functions. It was found that the fragile X mental retardation protein (FMRP) could bind to the G4s in its own mRNA coding region, thereby regulating its own translation through a negative feedback pathway [107]. Additionally, FMRP is likely to interact with G4s in other mRNAs for translation repression by the recruitment of translation inhibitors, miRNA pathway activation, and direct interaction with ribosomes [57]. FRAXE-associated mental retardation protein FMR2 could also bind to G4s in mRNAs and function in alternative splicing [108].

The rG4 in the region where proto-oncogene *NRAS* 5′-UTR folds into a stable intramolecular parallel G4 structure and it has been demonstrated that it represses translation in vitro [57]. The study revealed that DEAD box helicase DDX3X involved in several pathways of RNA biology could bind to *NRAS* rG4s and the mutations of DDX3X are associated with tumorigenesis, especially medulloblastoma [57]. In addition, some helicases such as DHX36 (DEAH-Box Helicase 36) and DDX21 are able to bind and unfold rG4 structures. Another multifunctional helicase DHX9 shows a binding affinity for several secondary nucleic acid structures including G4s, but it is more inclined to bind RNA substrates. Therefore, helicases with the function of recognition and resolution of rG4s may play essential roles in post-transcriptional biological processes such as mRNA translation, transportation and stability [46].

Although the vast majority of rG4s are present in mRNAs, others are also detected in long non-coding RNAs (lncRNAs) including nuclear paraspeckle assembly transcript 1 (*NEAT1*). *NEAT1* is involved in gene regulation as a scaffold for the assembly of paraspeckles [109]. An upregulation of *NEAT1* could be observed in the majority of solid tumors such as lung cancer, esophageal cancer and hepatocellular carcinoma, and *NEAT1* also plays a critical role in neurodegenerative diseases and viral infection [49,54]. Evidence has shown that nascent *NEAT1* transcripts interact directly with the non-POU domain-containing octamer-binding protein (NONO) through its conserved rG4 motifs. The primary paraspeckle formation is required for the recruitment of NONO to *NEAT1* transcripts which stabilizes *NEAT1* and lays the foundation for the recruitment of additional protein components to facilitate subsequent steps of assembly and maturation [54].

## 3. Structural Properties of G-Quadruplex-Binding Proteins

**Figure 4:**
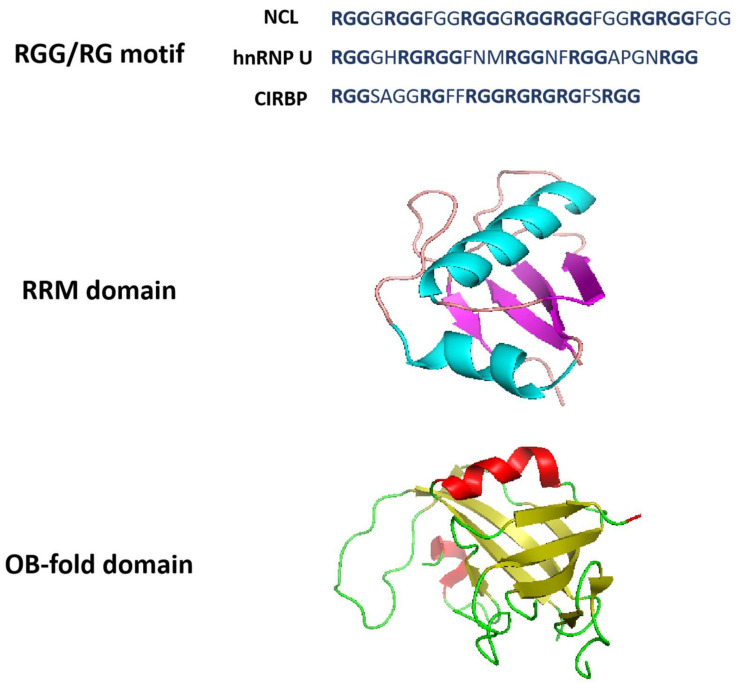
Structural properties of G-quadruplex-binding proteins. RGG/RG motifs are from NCL, hnRNP U and CIRBP. The RRM domain structure is derived from Protein Data Bank with structure code 2KRR (NCL) [112]. RRM domain is an αβ sandwich structure composed of one four-stranded antiparallel β-sheet and two α-helices packed against the β-sheet. The OB-fold domain structure is derived from Protein Data Bank with structure code 5W2L (CTC1) [113]. OB-fold domain is a β-barrel formed by five antiparallel β-sheets.

A comprehensive review revealed that the function of a protein is controlled by its amino acid sequence comprising domains and motifs [110]. The types of these domains are the basic elements that constitute the intrinsic properties of a protein such as interacting with nucleic acid sequences, and they also have an impact on the pathways to which it belongs [110]. The G4 recognition of proteins is a multistep process which involves the key domain recognizing the G4 structures through the interaction with adjacent disordered regions [111]. The analyses of known G4BPs suggest that the established or predicted binding regions in G4BPs have certain shared domains or motifs (Figure 4). Several studies indicate that the enrichment of these domains in G4BPs contributes to interacting with G4s [51,111]. The deeper insight into their characteristics will not only make progress in the binding mechanisms of G4-protein interactions, but also provide precise structural targets for subsequent drug design. Recent discoveries in the structural properties of G4BPs will be discussed in the following section.

### 3.1. RGG Domain

The RGG (Arginine-Glycine-Glycine) domain, also termed the RGG/RG motif or GAR (glycine-arginine-rich) domain is composed of repeat sequences rich in RGG or RG and is highly conserved in evolution (Figure 4) [110,111]. Researchers have discovered RGG/RG motifs in more than 1000 human proteins which influence transcription, precursor mRNA splicing, DNA damage signaling pathways, mRNA translation, and apoptosis [110]. A recent study analyzed the amino acid composition of 77 human G4-binding proteins [52]. Compared with a random subset of the human proteome and a well-defined group of nucleic acid binding proteins, the study demonstrated a significant enrichment of glycine and arginine and also high abundance in RR, GR and RG in G4BPs. Research was conducted to investigate the presence of a conserved RG-rich motif, which is a typical characteristic of G4BPs [52].

The RGG domain is usually found in G4BPs and it has been shown to mediate G4-protein interactions. For example, hnRNP U contains the RGG domain [62]. The C-terminal region of nucleolin composed of RNA-binding domain (RBD) 3 and 4 and the RGG domain is essential for the recognition of the *c-MYC* NHE III1 sequence and the promotion of G4 formation [60]. In addition, more than half of the newly identified *NRAS* rG4BPs contain the GAR domain which has been proved to be critical for *NRAS* rG4-DDX3X interaction [46].

The short residue gap between RGG repeats in the RGG domain frequently contains aromatic amino acids. The research on the binding mechanisms of the RGG domain revealed that the small segment RGG motif in this domain greatly contributes to the G4 binding affinity. Huang et al. found that the internal arrangement of RGG repeats and gap amino acids are more fundamental to G4-protein interactions than the length of RGG peptides and numbers of RGG repeats [60]. Experiments demonstrated that the peptide 12 with seven RGG repeats could efficiently bind to DNA G4s. Based on the above results, they discovered that the cold-inducible RNA-binding protein (CIRBP) containing peptide 12 could bind G4s both in vitro and in vivo, and this RGG peptide is essential for the G4 recognition of CIRBP [60]. The team provided a great deal of insight into the interaction between the RGG peptide and G4s, and identified a new G4-binding protein based on the exploration of G4-binding RGG motifs. In summary, this approach also adds a new dimension to the discovery of other G4BPs.

### 3.2. RRM Domain

Several G4BPs, such as hnRNPs, nucleolin, CIRBP, TLS/FUS (translocated in liposarcoma, also known as fused in sarcoma), and EWS (Ewing’s sarcoma), have shared structural features, such as RNA recognition motifs (RRM) and RGG domains [42]. RRM, also known as the RNA-binding domain (RBD) or ribonucleoprotein domain (RNP), is one of the most highly conserved nucleic acid binding domains that occurs in approximately 0.5–1% of human genes and folds into an αβ sandwich structure composed of one four-stranded antiparallel β-sheet and two α-helices packed against the β-sheet (Figure 4) [112,114,115]. Proteins with RRM are implicated in the regulation of transcription, translation, RNA processing, RNA export and stability [27], and they are also common in G4BPs.

The RRM and RGG domains at the C-terminal of nucleolin are necessary to inhibit and induce the formation of the G4 on the *c-MYC* promoter. The RRM in nucleolin can form G4s with guanine-containing single strands, but it unfolds G4s without guanines in the single strands of the 5′ and 3′ terminals [27]. The RRMs of hnRNP A1 and hnRNP D are able to bind and unfold G4s. The crystal structure of the two RRMs of hnRNP A1 with single-stranded telomeric DNA showed that RRM1 and RRM2 interact directly with d(TAGG) and d(TTAGG), respectively [116]. The RRM of hnRNP D could recognize d(TAG) in d(TTAGGG) determined by NMR [117]. A recent study indicated that a novel G4-binding protein SLIRP (stem-loop interacting RNA binding protein) also contains the RRM domain, which is required for efficient interaction between DNA G4s and SLIRP [118]. Furthermore, the sequence alignment for the RRMs derived from SLIRP and other G4BPs such as hnRNP A1 and nucleolin showed similar amino acid composition of these domains [118]. The findings of these studies shed light on the roles of the RRM domain conserved in many nucleic acid binding proteins and contribute greatly to the exploration of its biological functions.

### 3.3. OB-Fold Domain

Oligonucleotide/oligosaccharide binding (OB)-fold is a β-barrel structure comprising a five-stranded antiparallel β-sheet, and this barrel is capped by an α-helix located between the third and fourth strands (Figure 4) [119]. The OB-fold structure is highly dynamic, and the dynamic properties enable OB-fold containing proteins to participate in multiple cellular pathways, including the re-initiation of DNA synthesis and the maintenance of genome stability [120].

Replication protein A (RPA) is a single-stranded DNA-binding complex with three subunits which unfolds the G4s and is involved in various biological processes such as DNA replication, repair and recombination. Although both RPA and POT1-TPP1 can bind to telomeric overhangs, RPA is more abundant in cells [10]. The CST complex resembles RPA in that they harbor comparable arrays of OB-folds and possess small subunits with similar structures [34]. Since CST contains multiple OB-folds (one each in STN1 and TEN1, and seven in CTC1), it was estimated that CST could play distinct roles in replication using a dynamic binding mechanism similar to that observed in RPA [34,35,113]. The dynamic properties of RPA binding due to the microscopic dissociation and re-association of individual OB-folds allow RPA to diffuse along the single-stranded DNA and to melt unwanted DNA secondary structures [34]. In addition, POT1 also contains the OB-fold domain, and FRET has shown that it is critical for gradual G4 unfolding [73].

DHX36 can bind DNA and RNA G4 structures with high affinity. It is a multifunctional helicase involved in G4-dependent transcriptional and post-transcriptional regulation, and plays a critical role in heart development, hematopoiesis and embryogenesis in mice [121]. The DHX36-specific motif at the N-terminal of the protein forms a DNA-binding-induced α-helix that together with the OB-fold-like subdomain selectively binds to parallel G4s [121].

## 4. G-Quadruplex-Binding Proteins as Potential Drug Targets

### 4.1. Relationship between G-Quadruplex-Binding Proteins and Diseases

Multiple G4-recruited proteins functioning at G4s are critical for genome homeostasis since G4 structures pose a threat to genome stability by hindering efficient replication and inducing DNA damage. Genome instability caused by G4s is linked to several genetic disorders and may further contribute to carcinogenesis [122]. For instance, the elevated expression of G4-resolving helicases such as WRN and BLM has been demonstrated in cancer cells in order for highly proliferating cells to deal with increasing replicative lesions [53,123]. Aside from DNA helicases, a number of polymerases implicated in translesion synthesis (TLS) have the ability to replicate past G4 sites [53].

Similarly, various DNA repair proteins can interact with G4s, suggesting an important role of G4 structures in DNA repair pathways. It has been shown that G4 could positively or negatively affect DNA repair efficiency and the extent of the impact depends on the repair pathway itself [26,122]. Homologous recombination (HR) is critical for DSB (double-strand break) repair during DNA replication. It was proposed that G4 structures need to be processed by several HR factors such as Mre11 and DNA2 to initiate HR-mediated repair [124,125]. In addition, BRAC1 and BRAC2 are key proteins necessary for G4 modulation during HR and cancer cells with a deficient HR protein are sensitive to the treatment of G4-stabilizing ligands which leads to elevated DNA damage and cell death [122]. In the contrast, several studies indicate that G4 structures have positive effects on the nucleotide excision repair (NER) pathway that is frequently activated upon ultraviolent light (UV) irradiation and defects in NER may lead to a high risk of skin cancer development [122,126]. Recent experiments have demonstrated an increase in G4 formation after UV damage while the stabilization of G4s by the protein Zuo1 would further contribute to the recruitment of NER proteins and maintain genome integrity [127].

Additionally, G4BPs which regulate the expression of multiple proto-oncogenes influence gene transcription by stabilizing or unfolding the G4 structure on the promoters, thus they play an important role in cancer initiation and progression. For example, the G4 in *c-MYC* is the target of several G4BPs, such as nucleolin and NM23-H2, which are of great significance in cancer treatment. As a transcription activator of *c-MYC*, the decreased level of NM23-H2 will reduce the *c-MYC* expression, while it is also related to the enhancement of metastatic potential. In various cell types including lung epithelial cancer and A549 cells, decreased expression of *c-MYC* results in cell cycle arrest and apoptosis, and activation of apoptosis contributes greatly to a reduction in metastatic spread [43]. In summary, lower *c-MYC* levels caused by the decreased expression of NM23-H2 in cancer cells would diminish apoptosis of cancer cells and enhance metastatic potential as well [43]. Basically, a comprehensive study of the role that these proteins play in regulating the expression of genes required for tumorigenesis, maintenance and metastasis will enlighten novel cancer therapies by specifically altering their expression [10].

Secondly, several G4BPs are involved in the regulation of neurological diseases. For example, a notably decreased expression of FMRP that binds to G4-containing mRNA and regulates its transport could be observed in Fragile X syndrome, and consequently this reduction also directly affected the translation of several other mRNAs [128]. Another study showed that hnRNP H/F as important components of the cytoplasmic machinery responsible for the structural integrity of rG4 function in rG4-mediated translational control [129]. These proteins are an essential regulatory hub in glioblastoma (GBM) networks and hnRNP H/F overexpression in GBM drives translational control of rG4-containing mRNAs encoding proteins implicated in the maintenance of genome stability and the response to genotoxic damage [129]. Furthermore, an RNA binding protein GLN1 (Guanine Nucleotide-Binding protein-like 1) binds to the G4 structures in the 5’-UTRs of VPS35 and PRKN, and these two genes are related to Parkinson’s disease [130]. The target genes of an RNA G4-binding protein DDX3X encode proteins, thereby suggesting roles in the oxidative phosphorylation chain, while severe consequences and several diseases, including Huntington’s, Alzheimer’s, Parkinson’s disease and cancer are caused by dysregulation of the synthesis of oxidative phosphorylation components [46].

Researchers have also discovered that G4BPs play a role in the critical steps of viral infection. The presence of G4 has been confirmed in the genomes of SARS coronavirus [131], human papilloma [132], hepatitis C [133], Zika [134], Ebola virus and herpes simplex virus-1 (HSV-1) [59,135,136]. Human immunodeficiency virus-1 (HIV-1) which is the pathogeny of the acquired immune deficiency syndrome (AIDS) is regulated by G4 and its binding proteins. Researchers have identified functionally significant G4s in the Nef coding region and in the unique long terminal repeat (LTR) promoter of *HIV-1* [59]. The G4 formation in the LTR promoter region suppresses the transcription of *HIV-1*. Nucleolin strengthens inhibition capability by inducing G4 formation and stabilizing the G4 structure, while hnRNP A2/B1 activates *HIV-1* transcription by unfolding the LTR G4 structures [59]. Moreover, a recent study has demonstrated direct interaction between the G4 structure in the SARS-CoV-2 RNA genome and viral helicase nsp13. Targeting viral helicase and G4 structure is valuable for potentially inhibiting the SARS-CoV-2 virus [137].

### 4.2. Advances in G-Quadruplex-Binding Proteins for Drug Design

Since G4BPs are associated with various diseases, understanding the G4-protein interactions could provide new insight into therapeutic interventions when dysregulation of G4 formation and resolution has been considered as a pathogenic cause [138]. Moreover, G4s in live cells may exhibit polymorphism such as several conformations (folded or unfolded) available for ligand binding, such dynamic properties lead to the relatively poor specificity of G4-directed ligands. [139] Thus, researchers start to take into account G4-protein complexes as novel drug targets [55].

Several DNA helicases and polymerases which function in DNA repair pathways are involved in the resolution of G4s to prevent G4-driven genome instability. One clinical application is to design small-molecule helicase inhibitors for personalized cancer therapy as conventional approaches including chemotherapy and radiation would cause unpleasant side effects such as the cytotoxicity to normal cells and drug resistance [123]. Accordingly, a synthetic lethality approach could be exploited to combat cancer. The efficiency of this new therapy is usually predicated on the combination of a defective genetic background and an inhibitor of the DNA repair protein [53,123,140]. For example, PARP inhibitors have been applied to tumors characterized by a deficiency of BRAC1 or BRAC2 to enhance anticancer effects due to the vital role of the G4BP PARP1 in the DNA repair pathway [123]. Additionally, accumulating evidence shows that a helicase inhibitor in conjunction with G4 structure stabilization can also achieve synthetic lethality. For instance, the previous study demonstrated that cancer cells using a FANCJ-specific helicase inhibitor are highly sensitive to G4-stabilizing ligands [53,141]. Another promising clinical application is the introduction of G4-disrupting small molecules which can unfold G4s to rescue helicase impairment [142].

To lessen the side effects of conventional anticancer drugs, aptamers can be valuable for the development of efficient targeted drug delivery systems due to their high selectivity. Aptamers are short-folded oligonucleotides capable of specifically recognizing target molecules with high affinity. A unique option of aptamer architectures is G4 and G4-based aptamers have potential clinical applications. For example, the aptamer d(GGGT)_4_ able to form a 5’-5’ dimer of two stacked parallel G4s has shown anti-HIV activity by targeting the HIV-1 integrase (HIV-IN) [143]. Another 26-base G4 AS1411 aptamer which binds nucleolin on the cancer cell surface with high affinity and specificity has been widely used for cancer therapy and diagnosis [55,144]. AS1411 exhibits cancer-selective antiproliferative effects and causes cell death through its interaction with nucleolin [144].

In addition to the development of small-molecule inhibitors and G4-based aptamers, diverse techniques have been applied to target G4BPs. In recent years, proteolysis-targeting chimeras (PROTACs) which hijack the endogenous ubiquitin-proteasome system to degrade target proteins have emerged as a promising therapeutic modality for proteins including those non-druggable ones [138,145]. A novel strategy termed G4-PROTAC is generated from a combination of a G4 warhead and a E3 ligase recruiter. G4-PROTAC is designed for specific degradation of a G4-binding protein (RHAU/DHX36) which has been reported to be highly expressed in tissues of *C9orf72*-linked amyotrophic lateral sclerosis (ALS) patients and thus represents an important therapeutic target [138]. In conclusion, this approach has the prospect of extended application and holds great promise in potential therapeutics against diseases for which an aberrant expression of a G4-binding protein is known to be a major cause [138].

**Figure 5:**
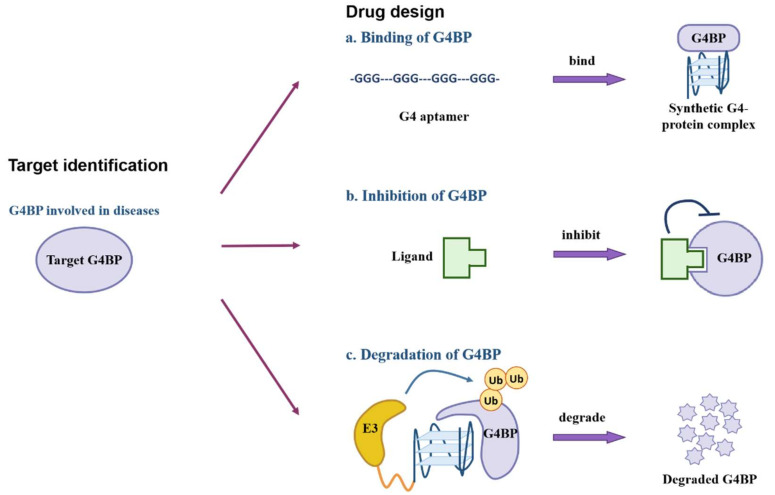
The schematic diagram for G-quadruplex-binding proteins as drug targets. The first step is target identification. After analyzing G4–protein interactions, G4BPs involved in disease-related pathways are chosen as potential drug targets. Secondly, three methods could be exploited for drug design. According to the G4–protein recognition mechanism, G4-based aptamers are designed to target specific proteins and form a synthetic G4–protein complex. Meanwhile, ligands would be used to inhibit G4BPs for synthetic lethality in cells with genetic defections. In addition, E3 ligase could be recruited for ubiquitin-mediated degradation of target proteins.

In summary, three kinds of methods can be exploited for drug design. First, G4 aptamers are designed to form a synthetic G4-protein complex for cellular function modulation. Second, small molecules or ligands are used to inhibit target proteins. Third, we could utilize the ubiquitin-proteasome system to degrade specific G4BPs (Figure 5). 

## 5. Conclusions and Perspectives

G4BPs are of vital importance in regulating the formation, stabilization and resolution of G4 structures in cells, and this regulatory mechanism has spatial and temporal properties. Generally, there are two kinds of classification methods for G4BPs. Firstly, we can divide these proteins into two main categories as follows: G4-folding proteins and G4-recruited proteins. Then, we can describe the control mechanisms of DNA and RNA G4BPs in detail according to the genomic distribution of G4s. Specifically, DNA G4BPs are mainly implicated in telomere maintenance, transcription, replication and epigenetic modification while RNA G4BPs are involved in mRNA maturation and translational regulation.

In recent years, a great number of new G4BPs have been identified, and researchers have also provided a good deal of insight into shared motifs and domains of these proteins. In terms of application, the targeting of G4BPs will serve as a breakthrough in drug innovation and add new dimensions to medical treatment. Owing to the prevalence of G4 motifs in the regulatory regions of the human genome, G4BPs play a vital role in different cellular pathways related to diseases and the dysregulation of G4BPs leads to tumor formation and viral infection. Nowadays, G4-resolving helicases and polymerases implicated in DNA repair pathways have been considered as a novel kind of drug targeting therapy with high medical value.

Subsequent studies will focus on the molecular mechanism of G4-protein recognition using high-throughput sequencing techniques and computational analyses [60,111]. We expect to have a more comprehensive understanding of the structural properties of G4BPs and the sequence features of their binding sites which lay the solid foundation for the identification of new G4BPs through G4-binding residues [52]. Furthermore, another research hotspot is the change in the regulatory mechanism of G4-protein interactions under normal physiological or abnormal pathological conditions. Since endogenous G4s and G4BPs are critical in the regulation of various biological processes, we ought to place emphasis on the improvement of current approaches targeting G4BPs [146]. Exogenous G4 aptamers present conformational polymorphisms which limit the characterization of their structural features, hence G4 aptamers are required to make modifications in order to overcome these structural drawbacks [146,147]. Meanwhile, bioinformatic research on G4BPs could unveil a deeper understanding of new target identification that offers clinical potential. The tremendous advances in computational modeling in the past decade have increased the speed and success rate of the discovery of small molecule drug candidates [148]. The DeepMind AlphaFold algorithm is a deep learning-based technology to accurately predict protein structures through amino acid sequences in the absence of experimental data, which provides an important structural basis for drug target discovery, drug design and molecular optimization [149,150]. New drug design targeting proteins requires structure-based virtual screening, so it is important to expand the scope of structure-based modeling. 

In conclusion, we expect to build a satisfactory database of G4BPs and collect the sequence-based features obtained from experiments, and then employ homology modeling and deep learning methods to predict the structures of these proteins [151]. On the basis of the relationship between G4BPs and diseases, we could screen potential proteins for drug target discovery and then design ligands and antibodies specific to target G4BPs for medical treatment. The integration of bioinformatics, structural biology, computational chemistry and machine learning will promote the development of novel, safe and effective drugs targeting G4BPs.

## Figures and Tables

**Table 1:** In this table, several key G4BPs with different biological functions are summarized. Their location, classification and structural features (if any) are listed.

Location	Biological Role	Protein Name	Classification	Structural Feature	PDB Code	References
DNA	Telomere maintenance	Shelterin complex	G4-recruited	OB-fold	3EDY	[31,32,33]
		CST complex	G4-recruited	OB-fold	5W2L	[34,35]
	G4 resolution and genome stability maintenance	WRN	G4-folding			[36,37]
		BLM				[38,39]
		FANCJ				[40,41]
	Transcriptional regulation	NCL	G4-folding	RGG; RRM	2KRR	[42]
		NM23-H2	G4-recruited			[43]
		p53				[44]
	Chromatin remodeling and DNA modification	ATRX	G4-recruited			[45]
		DNMT1				[28]
RNA	Translational regulation	DDX3X	G4-recruited	RGG	2JGN	[46,47]
		FMRP		RGG	5DEA	[48]
		NONO				[49]

## Data Availability

Not applicable.
